# 

**Published:** 1882-11

**Authors:** 


					﻿COLLABORATORS!
ALE. L. LOOMIS, M D......... New	York.
FESSENDEN N. OTIS, M D......
F. H. BOSWORTH, M.D.........
J. L. LITTLE, M.D...........
C. E. FRANCIS, D.D.S., M.D.S....
A. J. C. SKENE, M D....Brooklyn,	N.Y.
C. A. MARVIN, D.D.S......
W. C. BARRETT. M D.. D.D.S., Buffalo, “
EDWIN T. DARBY, M.D., D.D.S.... Phil., Pa.
P. S. WALES, M.D., Surg.-Gen’l U. S. Naw.
SAMUEL C. BUSEY, M.D.. Washington, D.C.
H. F. CAMPBELL, M.D......Augusta,	Ga.
C. H. MASTIN, M.D., LL.D...Mobile, Ala.
J. J. CHISOLM, M.D....Baltimore,	Md.
C. F. BEVAN. M.D........ “
I.	E. ATKINSON, M.D.....	“	“
H. McGUlRE. M.D.........Richmond, Va.
F. P. PORCHER, M D.....Charleston, S. C.
J.	W. UNDERHILL, M.D.....Cincinnati, O.
JAS. T. WHITTAKER, M.D....
J. RANSOHOFF, M.D., F.R.C.S.
F. FORCHHEIMER, M.D......
A. R. JACKSON. M.D........Chicago, Ill.
S. V. CLEVENGER, M.D...... “ “
E. F. INGALS, M.D......... “ “
J. H. CARSTENS, M.D......Detroit, Mich.
PROSPECTUS, 1882.
The Independent Practitioner is cos-
mopolitan in its scientific researches in
Medicine, Surgery, Obstetrics, Dentistry,
Hygiene, and Popular Science, and gives
the refined findings to its readers. It is
independent of colleges, cliques, isms, and
any advertising firm, and fearlessly renders
justice to all. Politics and religious creeds
are excluded.
				

